# Evaluation of Parameters for High Efficiency Transformation of *Acinetobacter baumannii*

**DOI:** 10.1038/srep22110

**Published:** 2016-02-25

**Authors:** Suleyman Yildirim, Mitchell G. Thompson, Anna C. Jacobs, Daniel V. Zurawski, Benjamin C. Kirkup

**Affiliations:** 1 Department of Wound Infections, Bacterial Diseases Walter Reed Army Institute of Research, Silver Spring, Maryland, USA; 2 Department of Medicine, Infectious Diseases Division, Uniformed Services University of the Health Sciences, F. Edward Hebert School of Medicine, Bethesda, Maryland, USA

## Abstract

*Acinetobacter baumannii* is an emerging, nosocomial pathogen that is poorly characterized due to a paucity of genetic tools and methods. While whole genome sequence data from several epidemic and environmental strains have recently become available, the functional characterization of genes is significantly lagging. Efficient transformation is one of the first steps to develop molecular tools that can be used to address these shortcomings. Here we report parameters allowing high efficiency transformation of *A. baumannii*. Using a multi-factorial experimental design we found that growth phase, voltage, and resistance all significantly contribute to transformation efficiency. The highest efficiency (4.3 × 10^8^ Transformants/μg DNA) was obtained at the stationary growth phase of the bacterium (OD 6.0) using 25 ng of plasmid DNA under 100 Ohms resistance and 1.7 kV/cm voltage. The optimized electroporation parameters reported here provide a useful tool for genetic manipulation of *A. baumannii.*

*Acinetobacter baumannii* is a gram negative, strictly aerobic, coccobacillus that has caused enormous public health concerns worldwide because of its remarkable ability to develop antibiotic resistance^1^. While the mortality rate of *A. baumannii*-associated disease can reach up to 68% in specific at-risk patient populations^2^, there are limited treatment options because of growing antibiotic resistance, and the pharmaceutical pipeline of novel compounds targeting this organism remains deficient^3^,^4^,^5^. The gap in *A. baumannii* drug development could be partly explained by the lack of genetic tools to functionally characterize genetic loci that contribute to pathogenesis. The multiple drug resistant (MDR) nature of relevant clinical isolates and incompatibility of many well-characterized plasmids commonly used for the molecular engineering of *Escherichia coli* make the genetic manipulation of *A. baumannii* a significant challenge^6^,^7^,^8^. As many previously discovered techniques used to introduce DNA into the *A. baumannii* genome rely on homologous recombination immediately after introduction into the cell, a high transformational efficiency is essential^9^,^10^. Though other work has employed electroporation as a method to transform *A. baumannii,* the optimization of transformation conditions for this bacterium has been underexplored^9^.

Electroporation is a method commonly used for introducing foreign DNA into cells across many phylogenies. Transformation efficiency in electroporation depends on multiple factors including: electrical parameters^11^, the amount and purity of DNA used, temperature, cell density, buffer composition, and the growth phase of the bacterial cells when made competent^12^. These factors must often be determined empirically to achieve optimal results^13^,^14^,^15^. Optimal transformation protocols are especially important for organisms with poorly defined or under developed genetic systems^16^ such as *A*. *baumannii*. Therefore, the aim of the present study was to evaluate parameters influencing transformation efficiency of *A. baumannii* using five different strains and a fractional factorial experimental design to achieve higher transformation efficiencies.

## Results and Discussion

### Experimental variables and the design of experiments (DOE)

To evaluate the effects of five variables (growth phase, voltage, resistance, plasmid DNA concentration, and the concentration of a divalent ion (magnesium)) on the transformation efficiency (Transformants/μg plasmid DNA) we used a three-level fractional factorial design to screen these factors in nine runs. Each experimental run was replicated three times to help identify sources of variation and to run an analysis of variance. The design matrix is shown in Table 1^ ^17,18 and the raw data collected are presented in the supplementary tables, Tables S1 and S2. We added a central point to the design matrix in addition to the low and high levels in order to analyze whether the level of each variable chosen led to improvement in the transformation efficiency. When the average effect size at the central point (Fave(0)) is smaller than the overall average of effect sizes at all other points (Fave(all)), the optimal point was localized toward the high-level settings of each variable. If it was greater, then the optimal point was located within the two-level variable settings^17^. The central point also allowed for evaluation of the relationship between each variable in the design and the response variable. When the average effect size at the center of the design was significantly different from the overall average of effect sizes at all other points the relationship between the variables and the response variable, it is likely to be non-linear^19^.

### Optimization runs of the electroporation conditions

**Figure 1:**
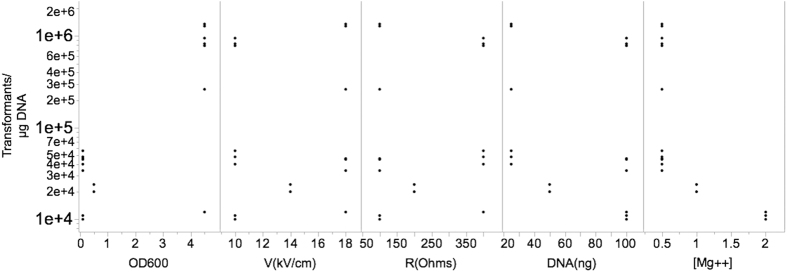
Scatter plot for DOE1. The response values (Transformants/μg DNA) at all levels of each factor are shown. The factors, growth phase, voltage, resistance, the amount of plasmid DNA used, and magnesium concentration are denoted by OD600, V (kV/cm), R (Ohms), DNA (ng), and [Mg++] mM, respectively.

**Figure 2:**
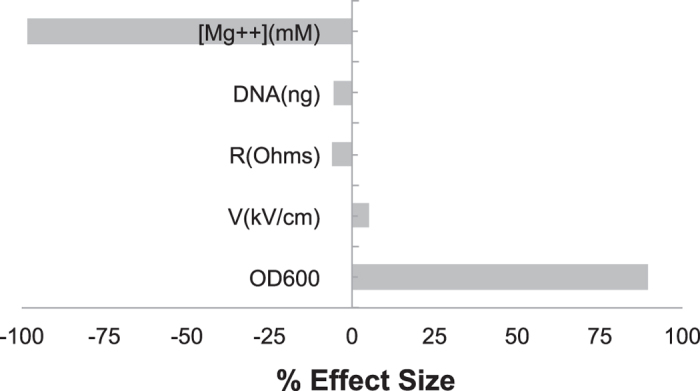
Percent effect size for each factor in DOE1. The Effect size difference between high level and low settings are divided by the average effect size of the entire experiment and expressed as percent effect size.

**Figure 3:**
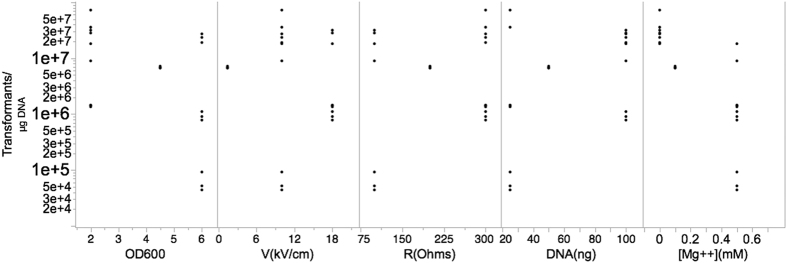
Scatter plot for DOE2. The response values (Transformants/μg DNA) at all levels of each factor are shown. The factors, growth phase, voltage, resistance, the amount of plasmid DNA used, and magnesium concentration are denoted by OD600, V (kV/cm), R (Ohms), DNA (ng), and [Mg++] mM, respectively.

**Figure 4:**
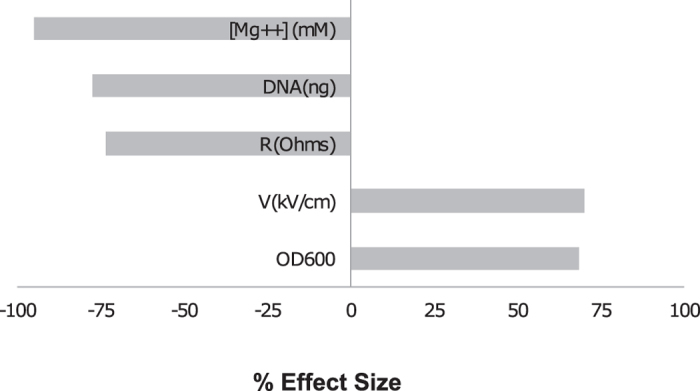
Percent effect size for each factor in DOE2. The Effect size difference between high level and low settings are divided by the average effect size of the entire experiment and expressed as percent effect size.

The DOE scatter plot in Fig. 1 (DOE1) shows the response values observed at all levels of each factor. The figure indicates trends in the location and scale for both within and between each variable, thereby helping to identify a ranked list of important variables contributing to transformation efficiency. The levels of two variables, OD_600_ (growth phase) and [Mg++] (concentration of magnesium), showed obvious location differences, suggesting that effect size at these levels significantly vary. Other variables did not fluctuate with great variation at both the low and high experimental levels. This could be due the levels chosen for these variables were not in a relevant range. Figure 2 summarizes the variations in percent average of effect sizes (Ef) for each factor based on DOE1. The variables, OD_600_ and [Mg++], clearly showed the highest percent Ef, 90% and −98%, respectively. The observed changes in other variables were as follows: 5% for V(kV/cm), −6% for R(Ohms), and −5% for DNA (ng). The Ef at central point was Fave(0) = 2.27 × 10^4^ and the overall Ef with other modified variables was Fave(all) = 2.4 × 10^5^. Since Fave(0) < Fave(all) the optimum of OD_600_ setting should be in “+1” level while [Mg++] should be localized in “−1” level. Thus, we reasoned that the updated levels of each variable, OD_600_ and [Mg++], are likely to significantly improve the overall transformation efficiency further. We therefore re-ran the transformation experiments again with three replicates utilizing the updated levels (see Table 1, footnotes). The scatter plot in Fig. 3 (DOE2) shows the response values at all levels of each variable at the updated levels. Unexpectedly, the variables, OD_600_ and resistance (R), showed distinct scale differences, and [Mg++] showed distinct location differences. Percent effect size variations caused by the high and low settings of these variables are summarized in the Fig. 4. The OD_600_ was 68%, V was 70%, R was (−73%), DNA was (−77%), and [Mg++] had (−95%). Additionally, Fave(all) = 6.95 × 10^7^ and Fave(0) = 6.89 × 10^6^. Fave(0) < Fave(all) in all the optimized settings of the variables are localized in lower settings of [Mg++], DNA, and R, and this is in contrast to V and OD_600_ variables. These results also suggested that the transformation efficiency is confounded by interactions of each variable.

We used JMP software (screening designs module) to fit a model to the data and identify significant interactions. Figure S1 shows a plot of actual versus predicted efficiency, a report of summary of fit, Analysis of Variance, and a prediction of desirability function. The model fits well as calculated by a high Rsquare, (*R*^*2 *^= 0.99) and (Prob > F*, p *< 0.0001). Significance of the regression parameters is determined by the Lenth t- Ratio statistics, calculated as Estimate/PSE, where PSE is Lenth’s Pseudo-Standard Error (see JMP DOE guide for more information on the description of the screening report: (https://www.jmp.com/support/downloads/pdf/jmp1001/doe_guide.pdf). The screening analysis identified the variables that have the most significant two-way interactions: [Mg++]*[Mg++], [Mg++]*DNA (ng), and DNA(ng)*V(kV). This result suggested that the effect of other two variables, OD_600_ and R (Ohms), are linear with transformation efficiency, that is, their effect is additive. The desirability function predicted parameter settings that maximizes transformation efficiency under the current model: when transformation is conducted under 100 Ohms, 18 V/cm, at late exponential phase (OD_600 _= 6.0) and with no magnesium ions present, then the predicted efficiency would be 4.44 × 10^8^ with the confidence intervals (4.3 × 10^8^ − 4.6 × 10^8^). Indeed, this prediction corresponds to the factor settings that resulted in maximum efficiency we obtained in transformation runs (Supplemental Table S2).

### Effect of growth phase (OD_600_) and validation of optimized conditions

**Figure 5:**
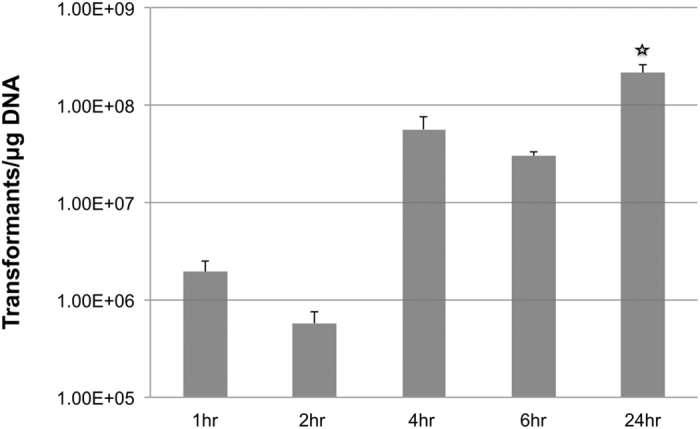
Transformation efficiency by growth phase. Variation of Transformation efficiency as a function of growth phase of *Acinetobacter baumannii,* AB5075. Error bars are obtained after replication of the transformation three times.

The effect size of growth phase (OD_600_) in DOE1 and DOE2 showed that the factor OD_600_ is the most important variable when compared to others, and model analysis indicated that this variable did not have a significant interaction with other parameters. Thus, we decided to investigate variation of the transformation only by growth phase. Interestingly, transformation efficiency at various phases of growth by the bacteria (Fig. 5) under the optimal conditions (the conditions that led to maximum efficiency and also predicted by the desirability function) showed that transformation efficiency significantly increased in stationary phase (24 hr point in Fig. 5; significance by *Tukey HSD* test; *p *< 0.001). This finding was unexpected as examples of high transformation efficiency of bacteria in stationary phase are highly uncommon. A recent report^20^ on the Gram negative species *Bacteroides fragilis,* demonstrated that the bacteria also had high transformation efficiency in stationary phase. Likewise, the Gram-positive species, *Corynebacterium pseudotuberculosis* and *Lactobacillus lactis* subsp. *lactis* were reported to have higher transformation efficiency in the stationary phase^21^,^22^. These two reports suggest that there are underlying mechanisms in common between both types of bacteria (Gram positive or negative) that play a role in high transformation efficiency in stationary phase. For example, membranes and/or the cell wall of both Gram-positive and Gram-negative bacteria maybe subjected to remodeling and/or turnover in response to nutrient limitation^23^, and this, in turn, may contribute to the increased efficiency of transformation in stationary phase. However, several other researchers^12^,^18^ reported that cell density in electroporation medium (Concentration Factor, CF) interact with the growth phase (OD) as OD*CF and, depending on the growth phase, this function evolves into an optimal point after which the transformation efficiency rapidly drops. Throughout this work we resuspended cells in 1.5 mL glycerol before electroporation from 50 mL growth medium, at a fixed CF, 33.3X. Because we did not include CF variable in the experimental design we did not look at OD*CF interaction in our experiments; thus we cannot rule out that the increased efficiency we observed in stationary phase could be due to OD*CF reaching optimal level and that there may be an optimal OD*CF in exponential phase that may yield high level efficiency.

**Figure 6:**
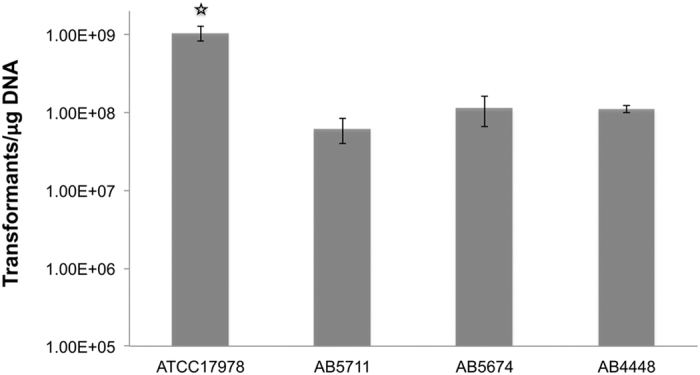
Strain-to-strain variation of transformation efficiency. Transformation efficiency of four strains of *Acinetobacter baumannii* under the optimized conditions. Error bars are obtained after replication of the transformation three times.

We validated the optimal conditions of electroporation by transforming four additional clinical strains of *A. baumannii* (Fig. 6), ATCC 17978, AB5711, AB5674, and AB4448. The transformation efficiency for these strains was as follows; 1.05 × 10^9^, 6.2 × 10^7^, 1.14 × 10^8^, and 1.11 × 10^8^, respectively, indicating that the optimized conditions are valid and consistent among various strains of the species. The reference strain (ATCC 17978) showed significantly higher transformation efficiency than other clinical strains possibly due to the exposure and adaptation to laboratory conditions over a long period of time.

In this study, we have demonstrated that the variables of electroporation to include: growth phase, voltage, resistance, and the amount of plasmid DNA as well as their two-way interactions significantly contribute to the transformation efficiency. Notably, magnesium, a divalent cation (Mg++), displayed an antagonistic effect in transformations when overall efficiency was compared. This effect was more pronounced when growth phase reached late exponential phase, and it is in contrast to previous findings with respect to Gram-negative bacteria as divalent cations have been shown to enhance transformation efficiencies. For example, the presence of cations enhances transformation in *E*. *coli*, and the absence of divalent cations reduces *E. coli* transformations up to 500-fold^24^. While the exact reason that [Mg++] decreases transformation efficiency remains unclear, it is known that [Mg++] bridges are thought to stabilize lipopolysaccharide (LPS), which is thought to be a permeability barrier associated with the outer membranes of Gram-negative bacteria^25^. The higher concentrations of [Mg++] may stabilize LPS of *A. baumannii*, which may limit transient pore formation during electroporation leading to reduced transformation efficiency, though further work needs to be done to test this hypothesis.

Although there may be other factors contributing to the transformation efficiency, such as restriction enzyme, temperature, washing buffer, concentration factor, and cell wall weakening agents^12^, the optimization of only four factors helped to increase the efficiency by four orders of magnitude with the highest transformation efficiency being 4.44 × 10^8^ transformant/μg DNA. This level of transformation frequency is usually more than sufficient for most downstream applications such as allelic exchange to develop knock out mutations and construction of mutant libraries for high-throughput screening.

The data presented here demonstrated that three-level fractional factorial designs (including center points) are effective in helping to screen several variables by substantially decreasing the number of total runs relative to full factorial designs. Indeed, a well-designed experiment should be cost-effective and enables the researcher to optimize conditions in the fewest possible experimental runs. The replication of each run while increasing the experimental cost did enable us to analyze variations and error in the experiments as well as interactions.

To conclude, our results indicate that high efficiency transformation of *A. baumannii* strains are obtained in stationary growth phase (OD 6.0) at a concentration factor 33.3X, and the presence of magnesium reduces the efficiency. The electroporation protocol conditions presented here should facilitate the genetic manipulation of *A. baumannii* strains, including the generation of knockout mutants, side-directed mutagenesis, heterologous gene expression, and random insertion mutagenesis using transposons.

## Materials and Methods

### Chemicals, Bacterial Strains, and Plasmids

Bacterial strains and the plasmid used in this study are listed in Table 2. *A. baumannii* strain AB5075^26^ and AB5711^27^ are clinical isolates and were used to optimize electroporation parameters. The *A. baumannii* clinical isolates AB4448 and AB5674, were obtained from Walter Reed Army Medical Center (WRAMC) and have been previously described and characterized^28^,^29^. The reference strain ATCC 17978 was obtained from the ATCC^30^. The *E. coli* strain DH10B was used to harbor plasmid pWH1266^31^. Selection for pWH1266 in both *E. coli* and *A. baumannii* was maintained with 10 μg/mL tetracycline (Sigma-Aldrich, St. Louis, MO). lysogeny (LB) Lennox Broth and agar plates (Becton, Dickinson and Co., Sparks MD) were used to maintain and propagate all strains; Super Optimal Broth (SOC) media (Sigma-Aldrich, St. Louis, MO) was used to recover cells post-pulse. Plasmid was isolated with the Zyppy™ Plasmid Miniprep Kit (Zymo Research, Irvine, CA), and all plasmids were eluted in 10 mM Tris EDTA (TE) at pH 8.0 and adjusted to a concentration of 50 ng/μL.

### Electroporation Procedure

A single colony of AB5075 from an LB plate was picked and grown overnight in 3 mL of LB media. The next day 0.5 mL of AB5075 media was inoculated into 50 mL of LB pre-warmed to 37 °C. Cells were grown at 37 °C with shaking at 250 rpm to the appropriate optical density (OD). Cells were pelleted at 10,000 x *g*, then washed twice with 25 mL of 10% glycerol at room temperature, and resuspended in a final volume of 1.5 mL of 10% glycerol. Electroporations were conducted using 50 μL of competent cells with 1 μL of the appropriate concentration of plasmid in 1 mm electroporation cuvettes (Eppendorf, Hamburg, Germany). All electroporations were carried out using a Bio-Rad GenePulser Xcell (Bio-Rad, Hercules, CA). After pulsing, the cells were immediately recovered in 1 mL of SOC media prewarmed to 37 °C, and then allowed to incubate at 37 °C for 1 hour. To enumerate post-pulse survival and transformants, cells were plated via spiral plater (Autoplate; Advanced Instruments, Inc., Norwood, MA) onto pre-warmed LB agar plates or LB agar plates supplemented with 10 μg/mL tetracycline, respectively. Plates were allowed to incubate overnight at 37 °C, and were then enumerated. Transformations were confirmed using plasmid preparations described above and enzymatic restriction enzyme digests performed with EcoRI, BamHI, etc. (Fermantas).

### Analyses of experimental data

We used JMP Statistical discovery from SAS (Cary, NC) software trial version (v.11) to plot the data and run model analysis using analysis of variance approach. The effect of a factor is the difference between average response at high level setting of a factor and average response at low setting of the same factor and can be mathematically calculated using the simple equation below (Antony, 2009):


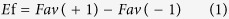


where Ef denotes the effect size of a factor, Fav (+1) is average effect size of the factor at high levels and Fav (−1) is average effect size of the factor at low levels.

## Additional Information

**How to cite this article**: Yildirim, S. *et al.* Evaluation of Parameters for High Efficiency Transformation of *Acinetobacter baumannii*. *Sci. Rep.* **6**, 22110; doi: 10.1038/srep22110 (2016).

## Supplementary Material

Supplemental Table S1 and S2

## Figures and Tables

**Table 1:** Matrix of Experimental Design.

Run#	OD600	V (kV/cm)	R (Ohms)	DNA (ng)	[Mg++] (mM)
1	+1	−1	+1	+1	−1
2	−1	−1	−1	+1	+1
3	+1	−1	−1	−1	+1
4	−1	−1	+1	−1	−1
5	−1	+1	+1	−1	+1
6	+1	+1	−1	−1	−1
7	−1	+1	−1	+1	−1
8	+1	+1	+1	+1	+1
9	0	0	0	0	0

DOE1 levels (−1, 0, +1): OD600 (0.1, 0.5, 4.5); V (10, 14, 18); R (100, 200, 400); DNA (25, 50, 100); Mg (0.5, 1, 2)

DOE2 levels (−1, 0, +1): OD600 (2, 4.5, 6); V (10, 14, 18); R (100, 200, 300); DNA (25, 50, 100); Mg (0.5, 1, 2)

**Table 2:** Bacterial strains and plasmid used in this study.

Strains and plasmid ID	Characteristics	References
AB5075	Clinical strain, Global Clonal Lineage I	Zurawski *et al.* 2012; Jacobs *et al.* 2014
ATCC17978	Reference strain	Baumann P, *et al.* 1968
AB5711	Clinical strainGlobal Clonal Lineage II	Zurawski *et al.* 2012; McQueary *et al.* 2012; Jacobs *et al.* 2014
AB5674	Clinical strainGlobal Clonal Lineage I	McQueary *et al.* 2012; Jacobs *et al.* 2014
AB4448	Clinical strainGlobal Clonal Lineage I	McQueary *et al.* 2012; Jacobs *et al.* 2014
pWH1266 (ATCC® 77092™)	Tet^R^, ampR, 8900 bp	Hunger *et al.* 1990
