# Supplementary material for: Evaluation of Parameters for High Efficiency Transformation of Acinetobacter baumannii
Source: Sci Rep. 2016 Feb 25;6:22110. doi: 10.1038/srep22110 (PMC4766488; doi:10.1038/srep22110)
Supplement: Supplemental Table S1 and S2 [file srep22110-s1.pdf]

Supplementary Information

Evaluation of Parameters for High Efficiency Transformation of *Acinetobacter baumannii*

Suleyman Yildirim[a*§], Mitchell G. Thompson[a], Anna C. Jacobs[a], Daniel V. Zurawski[a], Benjamin C. Kirkup Jr.[a,b¶],

[a] Department of Wound Infections, Bacterial Diseases Walter Reed Army Institute of Research, Silver Spring, Maryland, USA

[b] Department of Medicine, Infectious Diseases Division, Uniformed Services University of the Health Sciences, F. Edward Hebert School of Medicine, Bethesda, Maryland, USA

*Suleyman Yildirim: suleymanyildirim@medipol.edu.tr

[§]Present addresses:

Medipol University School of Medicine

Department of Medical Microbiology

Beykoz, Istanbul 34810

[¶]Center for Biomolecular Science and Engineering

Naval Research Labs,

4555 Overlook Ave SW, Washington DC 20375.

Supplemental Table S1. Transformation efficiency in replicate run of DOE1

Run#	OD600	V(kV/cm)	R (Ohms)	[DNA] ng	[Mg++] (mM)	Transformants/µg DNA	AVERAGE (n=3)	STDEV
1	4.5	10	400	100	0.5	7.83E+05	8.51E+05	8.61E+04
2	4.5	10	400	100	0.5	8.23E+05		
3	4.5	10	400	100	0.5	9.48E+05		
4	0.1	10	100	100	2	1.00E+04	1.03E+04	5.77E+02
5	0.1	10	100	100	2	1.00E+04		
6	0.1	10	100	100	2	1.10E+04		
7	4.5	10	100	25	2	0.00E+00	0.00E+00	0.00E+00
8	4.5	10	100	25	2	0.00E+00		
9	4.5	10	100	25	2	0.00E+00		
10	0.1	10	400	25	0.5	5.60E+04	4.80E+04	8.00E+03
11	0.1	10	400	25	0.5	4.80E+04		
12	0.1	10	400	25	0.5	4.00E+04		
13	0.1	18	400	25	2	0.00E+00	0.00E+00	0.00E+00
14	0.1	18	400	25	2	0.00E+00		
15	0.1	18	400	25	2	0.00E+00		
16	4.5	18	100	25	0.5	2.62E+05	9.65E+05	6.10E+05
17	4.5	18	100	25	0.5	1.35E+06		
18	4.5	18	100	25	0.5	1.28E+06		
19	0.1	18	100	100	0.5	3.40E+04	4.17E+04	6.66E+03
20	0.1	18	100	100	0.5	4.50E+04		
21	0.1	18	100	100	0.5	4.60E+04		
22	4.5	18	400	100	2	3.00E+02	4.10E+03	6.84E+03
23	4.5	18	400	100	2	0.00E+00		
24	4.5	18	400	100	2	1.20E+04		
25	0.5	14	200	50	1	2.00E+04	2.27E+04	2.31E+03
26	0.5	14	200	50	1	2.40E+04		
27	0.5	14	200	50	1	2.40E+04		

Supplemental Table S2. Transformation efficiency in replicate run of DOE2

Run#	OD600	V (kV/cm)	R (Ohms)	DNA (ng)	[Mg++] (mM)	Transformants/μg DNA	AVEAGE (n=3)	STDEV
**1**	6	10	300	100	0	1.91E+07	2.33E+07	4.11E+06
**2**	6	10	300	100	0	2.36E+07		
**3**	6	10	300	100	0	2.73E+07		
**4**	2	10	100	100	0.5	8.98E+06	1.21E+07	5.32E+06
**5**	2	10	100	100	0.5	1.82E+07		
**6**	2	10	100	100	0.5	8.98E+06		
**7**	6	10	100	25	0.5	5.20E+04	6.27E+04	2.57E+04
**8**	6	10	100	25	0.5	4.40E+04		
**9**	6	10	100	25	0.5	9.20E+04		
**10**	2	10	300	25	0	3.59E+07	4.82E+07	2.13E+07
**11**	2	10	300	25	0	7.28E+07		
**12**	2	10	300	25	0	3.59E+07		
**13**	2	18	300	25	0.5	1.35E+06	1.41E+06	5.11E+04
**14**	2	18	300	25	0.5	1.42E+06		
**15**	2	18	300	25	0.5	1.45E+06		
**16**	6	18	100	25	0	4.28E+08	4.44E+08	1.60E+07
**17**	6	18	100	25	0	4.44E+08		
**18**	6	18	100	25	0	4.60E+08		
**19**	2	18	100	100	0	2.83E+07	2.61E+07	7.06E+06
**20**	2	18	100	100	0	3.18E+07		
**21**	2	18	100	100	0	1.82E+07		
**22**	6	18	300	100	0.5	1.11E+06	9.33E+05	1.65E+05
**23**	6	18	300	100	0.5	9.05E+05		
**24**	6	18	300	100	0.5	7.83E+05		
**25**	4.5	14	200	50	0.1	7.20E+06	6.89E+06	2.80E+05
**26**	4.5	14	200	50	0.1	6.80E+06		
**27**	4.5	14	200	50	0.1	6.66E+06		
